# Comparative effects of ciprofol and propofol on perioperative outcomes: a systematic review and meta-analysis of randomized controlled trials

**DOI:** 10.1016/j.bjane.2024.844578

**Published:** 2024-11-26

**Authors:** Jiazheng Qi, Lingjing Zhang, Fanhua Meng, Xiaoyu Yang, Baoxuan Chen, Lingqi Gao, Xu Zhao, Mengqiang Luo

**Affiliations:** a Fudan University, Huashan Hospital, Department of Anesthesiology, Shanghai, China; b Sun Yat-sen University, The First Affiliated Hospital, Department of Anesthesiology, Guangzhou, China

**Keywords:** Ciprofol, Hemodynamics, Postoperative nausea and vomiting, Propofol, Respiratory insufficiency, Sedation

## Abstract

**Background:**

The ideal anesthetic agents for sedation, considering their respiratory and cardiovascular benefits and other perioperative or postoperative outcomes, are still unclear. This systematic review and meta-analysis aimed to evaluate whether ciprofol has advantages over propofol for sedation, particularly concerning respiratory and cardiovascular outcomes and other relevant perioperative measures.

**Methods:**

We conducted a comprehensive search of PubMed, Web of Science, the Cochrane Central Register of Controlled Trials, and two Chinese databases for randomized controlled trials comparing intravenous ciprofol and propofol for sedation. The primary outcome was the incidence of adverse respiratory events. Secondary outcomes included incidences of injection pain, hypotension, hypertension, bradycardia during surgery, perioperative nausea and vomiting, and postoperative awakening time. A random-effects model was used for more than four studies; otherwise, we employed the random-effects model with the Hartung-Knapp-Sidik-Jonkman adjustment.

**Results:**

Intravenous ciprofol resulted in fewer adverse respiratory events than propofol (Risk Ratio [RR = 0.44]; 95% Confidence Interval [95% CI 0.35–0.55], p < 0.001, I^2^ = 45%, low quality). It also showed a lower incidence of injection pain (RR = 0.12; 95% CI 0.08‒0.17, p < 0.001, I^2^ = 36%, low quality), intraoperative hypotension (RR = 0.64; 95% CI 0.52–0.77, p < 0.001, I^2^ = 58%, low quality), and nausea and vomiting than propofol (RR = 0.67; 95% CI 0.49–0.92; p = 0.01, I^2^ = 0%, moderate quality). However, no significant differences were observed for hypertension, bradycardia, and awakening time.

**Conclusions:**

Ciprofol may be more effective than propofol in minimizing perioperative respiratory adverse events and maintaining hemodynamic stability during sedation without prolonging recovery time.

## Introduction

Propofol is the most commonly used agent for sedation,^1^ largely due to its rapid onset of action, short half-life, quick metabolism, association with minimal complications, and short recovery time.^2^ However, it may not be suitable for all patients due to potential drawbacks, including dose-dependent respiratory depression, significant hemodynamic changes, and potential adverse effects such as injection pain.^3^, ^4^, ^5^, ^6^

Ciprofol is a newly developed drug which is structurally similar to propofol.^7^ It binds to the α1β2γ2 subtype of the gamma-aminobutyric acid-A receptor and offers improved pharmacological and physicochemical properties compared to propofol. Phase I–III trials conducted in China and Australia indicate that 0.2–0.5 mg.kg^-1^ of ciprofol provides comparable sedation levels, recovery times, or quality to 2.0 mg.kg^-1^ of propofol. Additionally, ciprofol has demonstrated effectiveness in gastrointestinal endoscopy and intensive care unit sedation, with lower incidences of hypotension and bradycardia than propofol.^8^ However, the evidence regarding the use of ciprofol for sedation needs to be collated and evaluated to determine its suitability as an alternative.

In this systematic review and meta-analysis, we aimed to compare the effectiveness and safety of ciprofol and propofol for sedation and summarize the effect of intravenous ciprofol on adverse respiratory events, hemodynamics, and recovery after sedation based on Randomized Controlled Trials (RCTs).

## Methods

### Study design

This study was conducted and reported in accordance with the Preferred Reporting Items for Systematic Reviews and Meta-Analyses statement.^9^ A predefined protocol was prospectively registered in the International Prospective Registry of Systematic Reviews on October 27, 2023 (PROSPERO CRD42023472833).

### Search strategy and selection criteria

We systematically searched for relevant studies in the following databases: PubMed, Web of Science, the Cochrane Central Register of Controlled Trials, and two Chinese databases (Weipu and Wanfang). The search was conducted from the inception of these databases until November 27, 2023. We also searched the reference lists of identified review articles for additional trials that were not initially identified in our electronic searches of the primary databases. To strengthen the comprehensiveness and scope of the application of the included studies, no limitations were imposed on the age of the individuals included in the analysis, and no language restrictions were applied. Since the literature on ciprofol is scarce, our search strategy focused exclusively on the term “ciprofol” (Appendix 1 of the Supplementary Files).

We included trials that compared intravenous ciprofol and propofol in patients undergoing sedation. This intervention could involve the use of intravenous ciprofol alone or in combination with other sedatives (e.g., benzodiazepines) or analgesics (e.g., opioids). Propofol alone or in combination with other sedatives or analgesics was used as the comparator. Exclusion criteria were as follows: studies that did not compare ciprofol with propofol, those involving patients classified as American Society of Anesthesiologists (ASA) physical status classification IV–V, non-elective surgeries, the use of muscle relaxants or mechanical ventilation, or studies with incomplete data. Two authors independently screened the titles and abstracts to identify eligible full-text articles. Discrepancies regarding trial inclusion were resolved through consultation with a third author.

Data extraction was performed independently by two authors using a standard data collection template. This template included information on the authors, publication date, type of surgery, intervention group, control group, dosage of each anesthetic, and outcome data.

### Measurement of outcome data

The primary outcomes were adverse respiratory events, which included hypoxemia, airway obstruction, apnea, laryngospasm, and airway interventions (such as chin lift/chin push, increased oxygen flow, and assisted ventilation). In contrast, the secondary outcomes included injection pain, hypotension, hypertension, bradycardia, nausea and vomiting, and postoperative awakening time. Definitions for secondary outcomes were based on those provided in the individual studies. Specifically, time to awakening was defined as the duration required to achieve three consecutive modified Observer's Assessment of Alertness/Sedation scores > 5 postoperatively.^10^^,^^11^ Perioperative nausea and vomiting referred to any instances of nausea or vomiting occurring throughout the sedation and recovery periods.

### Assessment of the risk of bias

Using the Cochrane Collaboration tool for randomized trials (Cochrane Handbook for Systematic Reviews of Interventions), two reviewers independently assessed the Risk of Bias (RoB) concerning the randomization process, potential bias from the intended intervention, the completeness of outcome data, risks associated with outcome measurement, and the selection of reported outcomes.

The quality of evidence was evaluated independently by two reviewers using the Grading of Recommendations Assessment, Development and Evaluation system, which considers the following five criteria: RoB, inconsistency, indirectness, imprecision, and publication bias. Discrepancies in the assessments were resolved through discussion or by consulting a third author if necessary.

### Data synthesis and analysis

Data from each of the included studies were extracted and integrated into data tables by two independent researchers. Data synthesis was conducted using Review Manager 5.3 software (version 5.3; The Nordic Cochrane Center, The Cochrane Collaboration, Copenhagen, Denmark), and Hartung-Knapp-Sidik-Jonkman (HKSJ) adjusted random-effects model was performed using R software (version 4.4.0).

Statistical heterogeneity was assessed using the I^2^ statistic, which quantifies the percentage of total variability between studies attributed to heterogeneity rather than chance. The degree of heterogeneity was evaluated according to the Cochrane Handbook guidelines.^12^ A random-effects model was used to synthesize the data. However, when fewer than five studies are included in a meta-analysis, it is typically regarded as a small-size meta-analysis, for which traditional random-effects models may be inappropriate. Therefore, employing the HKSJ-adjusted random-effects model is recommended to ensure the robustness of the results.^13^^,^^14^ The mean difference, 95% Confidence Interval (95% CI), and p-value were reported for continuous outcomes, whereas the Risk Ratio (RR), 95% CI, and p-value were recorded for categorical outcomes.

Statistical significance was set at p < 0.05. If a sufficient number of publications was available (n = 10), publication bias was assessed via funnel plots (visually) and more formally with the Egger's test.^15^ Additionally, to explore sources of heterogeneity, “leave-one-out” sensitivity and subgroup analyses were conducted for the primary outcomes.

## Results

**Figure 1:**
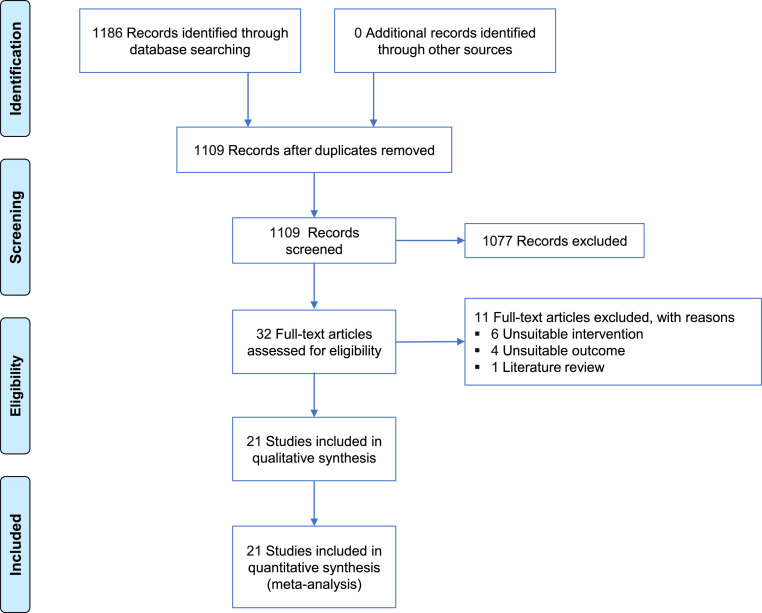
Flow diagram of the study selection process.

Figure 1 shows the flowchart of the study selection process. Initially, 1,186 relevant citations were identified. After removing duplicates, 1,109 citations remained. Following title and abstract screening, 32 full-text articles were selected for further assessment. After excluding one literature review,^16^ 6 articles without the required interventions,^17^, ^18^, ^19^, ^20^, ^21^, ^22^ 4 lacking essential outcome information,^23^, ^24^, ^25^, ^26^ and 21 RCTs were included in the final analysis.^10^^,^^11^^,^^27^, ^28^, ^29^, ^30^, ^31^, ^32^, ^33^, ^34^, ^35^, ^36^, ^37^, ^38^, ^39^, ^40^, ^41^, ^42^, ^43^, ^44^, ^45^ Among the included studies, 15 (with data from 2,878 patients) focused on painless gastrointestinal surgery.^10^^,^^27^, ^28^, ^29^^,^^32^, ^33^, ^34^, ^35^, ^36^, ^37^, ^38^^,^^40^, ^41^, ^42^^,^^44^ Two studies (involving 192 patients) investigated painless fibrobronchoscopy.^30^^,^^39^ Additionally, two studies (with 285 patients) reported painless hysteroscopy.^31^^,^^43^ One study (based on 207 patients) examined non-operating room procedures, including endoscopic submucosal dissection, endoscopic retrograde cholangiopancreatography, and fibrobronchoscopy.^11^ Furthermore, one article (including 284 patients) reported on painless endoscopic retrograde cholangiopancreatography.^45^ Detailed characteristics of the studies are presented in Table 1.

**Table 1:** Characteristics of the included studies.

Study	n	Surgery	Treatment group	Controlled group	Respiratory adverse event
Zhong J 2023^11^	207	ESD, ERCP, or fibrobronchoscopy	a)6 mg.kg^-1^.h^-1^ ciprofol for induction; 1‒2.5 mg.kg^-1^.h^-1^ ciprofol for maintenance	40 mg.kg^-1^.h^-1^ propofol for induction, 5‒12.5 mg.kg^-1^.h^-1^ propofol for maintenance	Hypoxemia
			b) 8 mg.kg^-1^.h^-1^ ciprofol for induction; 1‒2.5 mg.kg^-1^.h^-1^ ciprofol for maintenance		
			ESD: combined with 1.5 ng.mL^-1^ remifentanil and maintained 0.1‒0.5 ng.mL^-1^ remifentanil	ESD: combined with 1.5 ng.mL^-1^ remifentanil and maintained 0.1‒0.5 ng.mL^-1^ remifentanil	
			ERCP: combined with 0.2 mg.kg^-1^ esketamine		
				ERCP: combined with 0.2 mg.kg^-1^ esketamine	
			Fibrobronchoscopy: combined with 4 ng.mL^-1^ remifentanil and maintained 0.5‒1 ng.mL^-1^ remifentanil	Fibrobronchoscopy: combined with 4 ng.mL^-1^ remifentanil and maintained 0.5‒1 ng/mLremifentanil	
Li J 2022^10^	289	Gastroscopy or colonoscopy	0.4 mg.kg^-1^ ciprofol + 50 μg fentanyl, if mOAA/S > 1 after 2 min of initial administration, infuse 1/2 initial dose of ciprofol	1.5 mg.kg^-1^ propofol + 50 μg fentanyl, if mOAA/S > 1 after 2 min of initial administration, infuse 1/2 initial dose of propofol	Hypoxemia; respiratory depression; apnea
Wu B 2022^39^	92	Fiberoptic bronchoscopy	0.3 mg.kg^-1^ ciprofol + 50 μg fentanyl, if required, added 1/3 or 1/4 initial dose of ciprofol	1.2 mg.kg^-1^ propofol + 50 μg fentanyl, if required, added 1/3 or 1/4 initial dose of propofol	Hypoxemia; respiratory depression
Lan H 2023^31^	149	Hysteroscopy	0.4 mg.kg^-1^ ciprofol + 0.1 μg.kg^-1^ sufentanil, added 0.6‒1.2 mg.kg^-1^.h^-1^ ciprofol to maintain BIS value between 40‒69	2.0 mg.kg^-1^ propofol + 0.1 μg.kg^-1^ sufentanil, added 3.0‒6.0 mg.kg^-1^.h^-1^ propofol to maintain BIS value between 40‒69	Hypoxemia; apnea
Zhao W 2023^45^	284	ERCP	0.3‒0.4 mg.kg^-1^ ciprofol + 0.1 μg.kg^-1^ sufentanil, maintained at 1.0‒1.5 mg.kg^-1^.h^-1^ ciprofol	1.5‒2.0 mg.kg^-1^ propofol + 0.1 μg.kg^-1^ sufentanil, maintained at 4‒12 mg.kg^-1^.h^-1^ propofol	Hypoxemia
Zhang Xiang 2023^44^	100	Painless gastroscopy	0.3 mg.kg^-1^ ciprofol + 0.1 μg.kg^-1^ sufentanil, 0.075 mg.kg^-1^ ciprofol if needed	1.5 mg.kg^-1^ propofol + 0.1 μg.kg^-1^ sufentanil, 0.375 mg.kg^-1^ propofol if needed	Hypoxemia; respiratory depression
Zhang Xiao 2023^43^	136	Hysteroscopy	0.4 mg.kg^-1^ ciprofol for induction, added 0.6‒1.2 mg.kg^-1^.h^-1^ ciprofol + 1.5 μg.mL^-1^ remifentanil	2 mg.kg^-1^ propofol for induction, added 3‒6 mg.kg^-1^.h^-1^ propofol + 1.5 μg.mL^-1^ remifentanil	Respiratory depression
Zhang J 2023^42^	185	Gastrointestinal endoscopy	0.3 mg.kg^-1^ ciprofol + 0.7 mg.kg^-1^ alfentanil, added 5 mg ciprofol if needed	1.2 mg.kg^-1^ propofol + 0.7 mg.kg^-1^ alfentanil, added 20 mg propofol if needed	Respiratory depression
Yi Q 2022^41^	159	Gastroscopy	0.2 mg.kg^-1^ ciprofol + 0.1 μg.kg^-1^ sufentanil, added 0.2 mg.kg^-1^ ciprofol if needed	1 mg.kg^-1^ propofol + 0.1 μg.kg^-1^ sufentanil, added 0.5 mg.kg^-1^ propofol if needed	Respiratory depression
Xu M 2023^40^	322	Fiberoptic bronchoscopy	0.4 mg.kg^-1^ ciprofol + 0.1 μg.kg^-1^ sufentanil, if needed, added 1‒1.5 mg.kg^-1^.h^-1^ ciprofol	2 mg.kg^-1^ propofol + 0.1 μg.kg^-1^ sufentanil, if needed, added 4‒6 mg.kg^-1^.h^-1^ propofol	Hypoxemia
Wang J 2023^38^	100	Gastrointestinal endoscopy	0.4 mg.kg^-1^ ciprofol + 0.2 mg.kg^-1^ esketamine, if needed, added 5 mg ciprofol	2 mg.kg^-1^ propofol + 0.2 mg.kg^-1^ esketamine if needed, added 0.3‒0.5 mg.kg^-1^ propofol	Respiratory depression
Wang C 2023^37^	100	Colonoscopy	0.2 mg.kg^-1^ ciprofol + 5 μg.kg^-1^ alfentanil, added 0.1 mg.kg^-1^ ciprofol if needed	1 mg.kg^-1^ propofol + 5 μg.kg^-1^ alfentanil, added 0.5 mg.kg^-1^ propofol if needed	Hypoxemia
Liu XY 2023^36^	350	Gastroscopy	0.2 mg.kg^-1^ ciprofol + 0.1 μg.kg^-1^ sufentanil	1 mg.kg^-1^ propofol + 0.1 μg.kg^-1^ sufentanil	Respiratory depression
Liu X 2023^35^	100	Gastrointestinal endoscope	0.5 mg.kg^-1^ ciprofol + 4 μg.kg^-1^ alfentanyl, if needed, added 0.125 mg.kg^-1^ ciprofol	2 mg.kg^-1^ propofol + 4 μg.kg^-1^ alfentanyl, if needed, added 0.5 mg.kg^-1^ propofol	Respiratory depression
Liu S 2023^34^	280	Gastroscopy	0.5‒0.6 mg.kg^-1^ ciprofol, if needed, added 5 mg ciprofol	1.5 mg.kg^-1^ propofol + 5 μg.kg^-1^ remifentanil, if needed, added 20 mg propofol	Hypoxemia; respiratory depression
Liao J 2023^33^	368	Gastrointestinal endoscopy	0.4 mg.kg^-1^ ciprofol + 0.05 μg.kg^-1^ sufentanil	2 mg.kg^-1^ propofol + 0.05 μg.kg^-1^ sufentanil	Hypoxemia; apnea
Liang W 2023^32^	159	Gastroscopy	(a) 0.4 mg.kg^-1^ ciprofol, if needed, add 0.1 mg.kg^-1^ ciprofol	2 mg.kg^-1^ propofol, if needed, add 2 mg.kg^-1^ propofol	Hypoxemia
			(b) 0.5 mg.kg^-1^ ciprofol, if needed, add 0.1 mg.kg^-1^ ciprofol		
			(c) 0.6 mg.kg^-1^ ciprofol, if needed, add 0.1 mg.kg^-1^ ciprofol		
Huang X 2023^30^	100	Flexible bronchoscopy	0.4 mg.kg^-1^ ciprofol + 0.1 μg.kg^-1^ sufentanil, if needed, added 5 mg ciprofol	1.5 mg.kg^-1^ propofol + 0.1 μg.kg^-1^ sufentanil, if needed, added 20 mg propofol	Hypoxemia; Respiratory depression
Gao Z 2023^29^	121	Gastroenteroscopy	0.3‒0.4 mg.kg^-1^ ciprofol + 0.1 mg.kg^-1^ nalbuphine, if needed, added 1/2 initial ciprofol	1.2‒1.6 mg.kg^-1^ propofol + 0.1 mg.kg^-1^ nalbuphine, if needed, added 1/2 initial propofol	Respiratory depression
Chen X 2022^28^	96	Gastroenteroscopy	0.4 mg.kg^-1^ ciprofol for induction	1.5 mg.kg^-1^ propofol for induction	Not reported
Chen L 2023^27^	149	Gastrointestinal endoscopy	(a) 0.2 mg.kg^-1^ ciprofol + 2 μg.kg^-1^ fentanyl	1.5 mg.kg^-1^ propofol + 2 μg.kg^-1^ fentanyl	Respiratory depression
			(b) 0.3 mg.kg^-1^ ciprofol + 2 μg.kg^-1^ fentanyl		
			(c) 0.4 mg.kg^-1^ ciprofol + 2 μg.kg^-1^ fentanyl		

ESD, Endoscopic Submucosal Dissection; ERCP, Endoscopic Retrograde Cholangiopancreatography; mOAA/S, modified Observer's Assessment of Alertness/Sedation scale.

**Figure 2:**
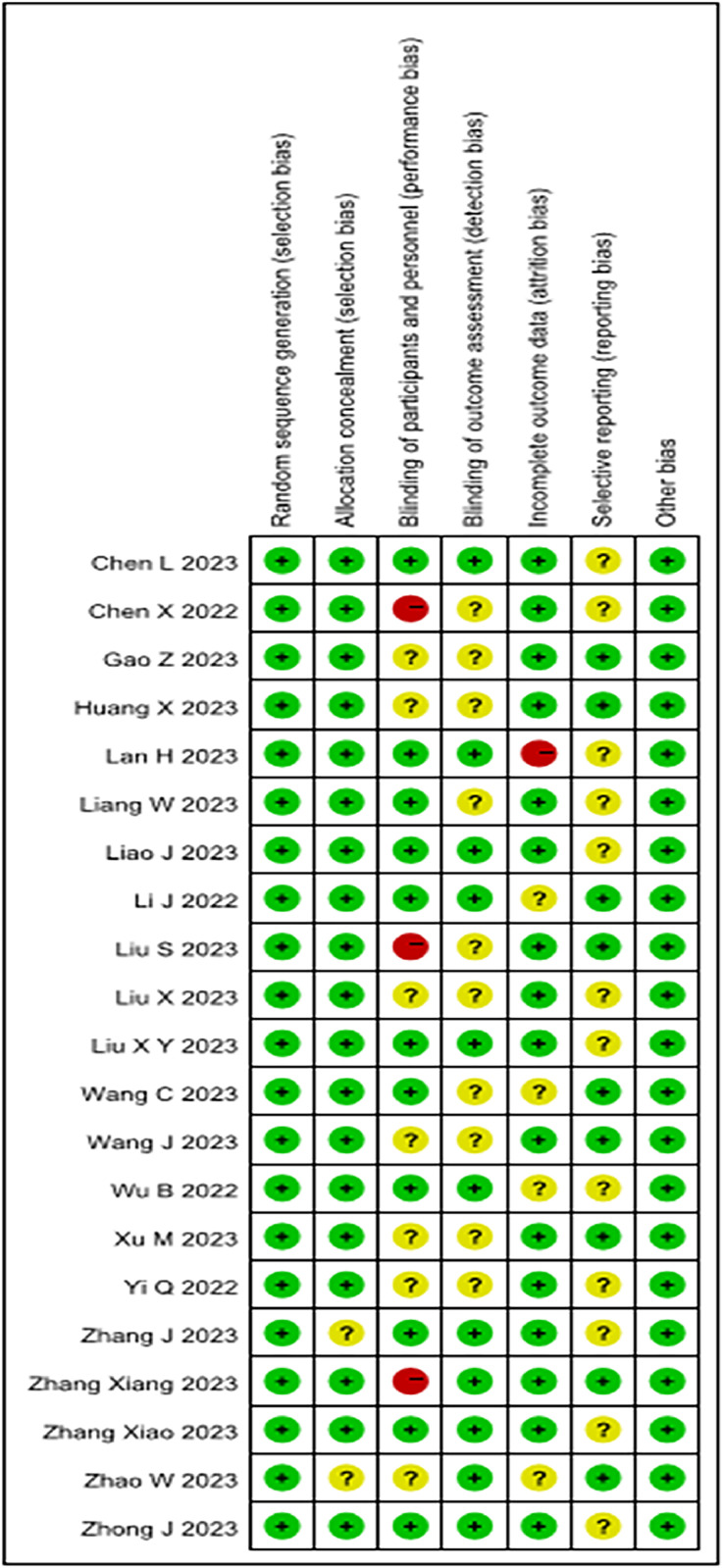
Risk of bias summary (green: low; yellow: unclear; red: high).

The RoB was assessed as shown in Figure 2. Of the 21 studies selected, 14 were categorized as having low RoB, 8 failed to clearly describe the procedures for implementing blinding, including not specifying who was blinded or providing sufficient details about the blinding process, which could have increased the differences between the two groups,^28^, ^29^, ^30^^,^^34^^,^^35^^,^^41^^,^^44^^,^^45^ and 1 had unclear results,^31^ which impacted the quality of reporting and overall literature (Supplementary Table 1).

### Primary outcomes: adverse respiratory events during sedation

**Figure 3:**
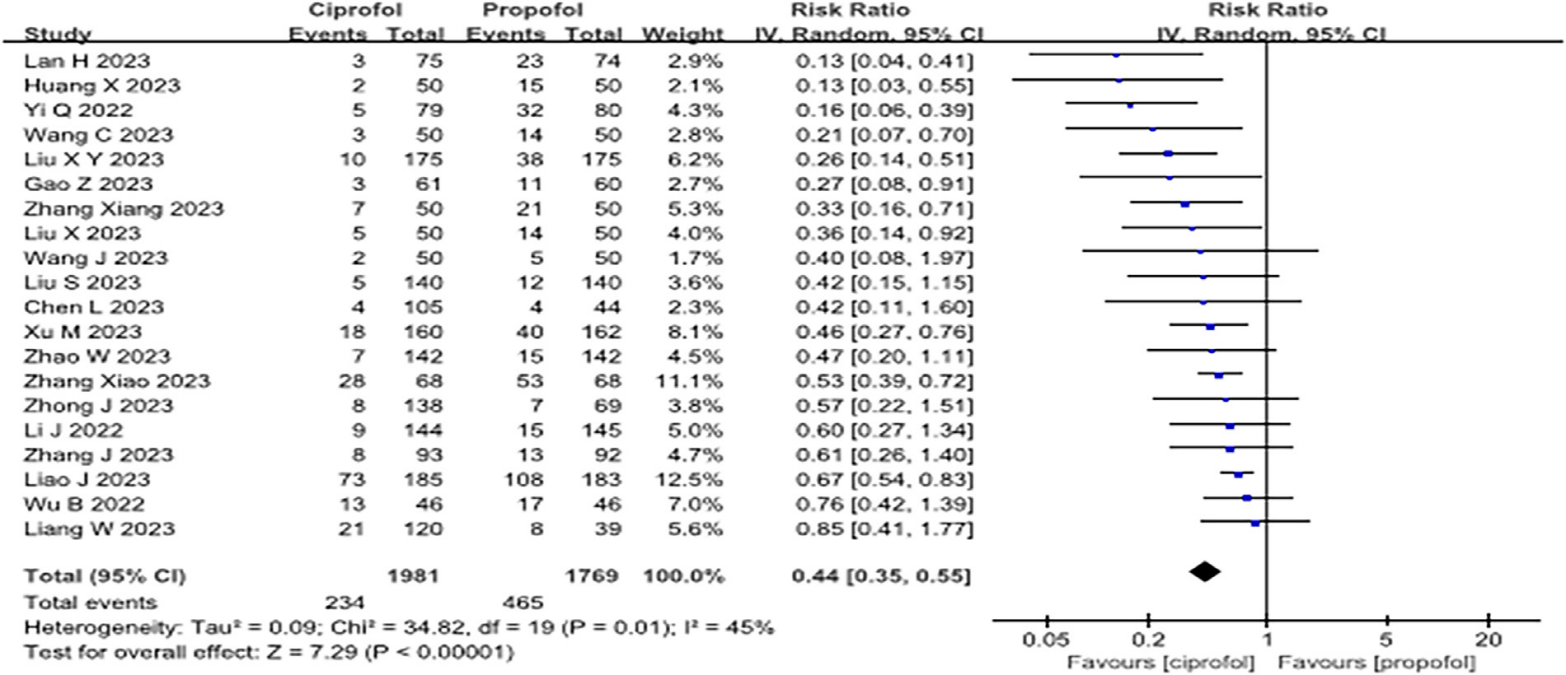
Forest plot of the incidence of adverse respiratory events during sedation. "IV, Random" indicates that the inverse variance method under a random-effects model was used. When the number of included studies exceeded 4, this traditional random-effects model was applied to pool effect sizes.

Twenty articles^10^^,^^11^^,^^27^^,^^29^, ^30^, ^31^, ^32^, ^33^, ^34^, ^35^, ^36^, ^37^, ^38^, ^39^, ^40^, ^41^, ^42^, ^43^, ^44^, ^45^ have reported the effects of ciprofol on adverse respiratory events during sedation. Compared to propofol, intravenous administration of ciprofol significantly reduced the incidence of adverse respiratory events (RR = 0.44; 95% CI 0.35–0.55, p < 0.001, I^2^ = 45%, low quality, Fig. 3 and Supplementary Table 1). Publication bias assessed using funnel plots indicated potential bias. Additionally, 70% of the studies included for this outcome were classified as high-risk, which contributed to the low-quality rating of the evidence. Sensitivity analyses were performed by sequentially excluding each of the 20 articles. None of these exclusions affected the overall results, as detailed in Supplementary Table 2. Subgroup analyses based on the type of opioids (short- or long-acting) used in combination with the sedatives and the type of surgery are presented in Supplementary Figures 7 and 8, respectively. These analyses suggested that the type of opioids and surgery might be a source of heterogeneity. Furthermore, after excluding studies with a high RoB, the significance of the results remained unchanged, as illustrated in Supplementary Figure 2.

### Secondary outcomes

#### Injection pain

**Figure 4:**
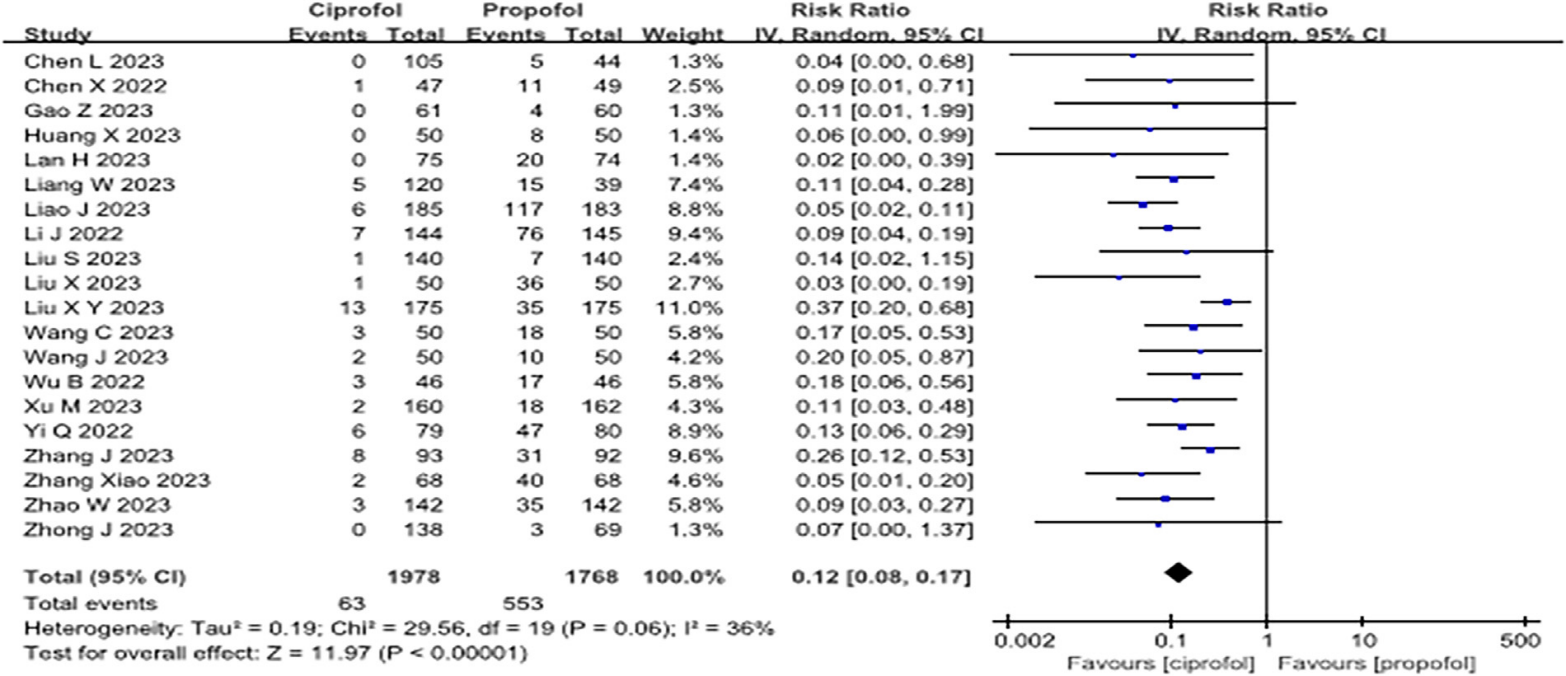
Forest plot of the incidence of injection pain during sedative sedation. "IV, Random" indicates that the inverse variance method under a random-effects model was used. When the number of included studies exceeded 4, this traditional random-effects model was applied to pool effect sizes.

Twenty articles^10^^,^^11^^,^^27^, ^28^, ^29^, ^30^, ^31^, ^32^, ^33^, ^34^, ^35^, ^36^, ^37^, ^38^, ^39^, ^40^, ^41^, ^42^, ^43^^,^^45^ documented the effect of ciprofol on the occurrence of injection pain. Patients showed a markedly decreased incidence of injection pain during anesthesia induction with ciprofol compared to propofol (RR = 0.12; 95% CI 0.08–0.17, p < 0.001, I^2^ = 36%, low quality, Fig. 4 and Supplementary Table 1).

#### Hypotension, hypertension, and bradycardia

**Figure 5:**
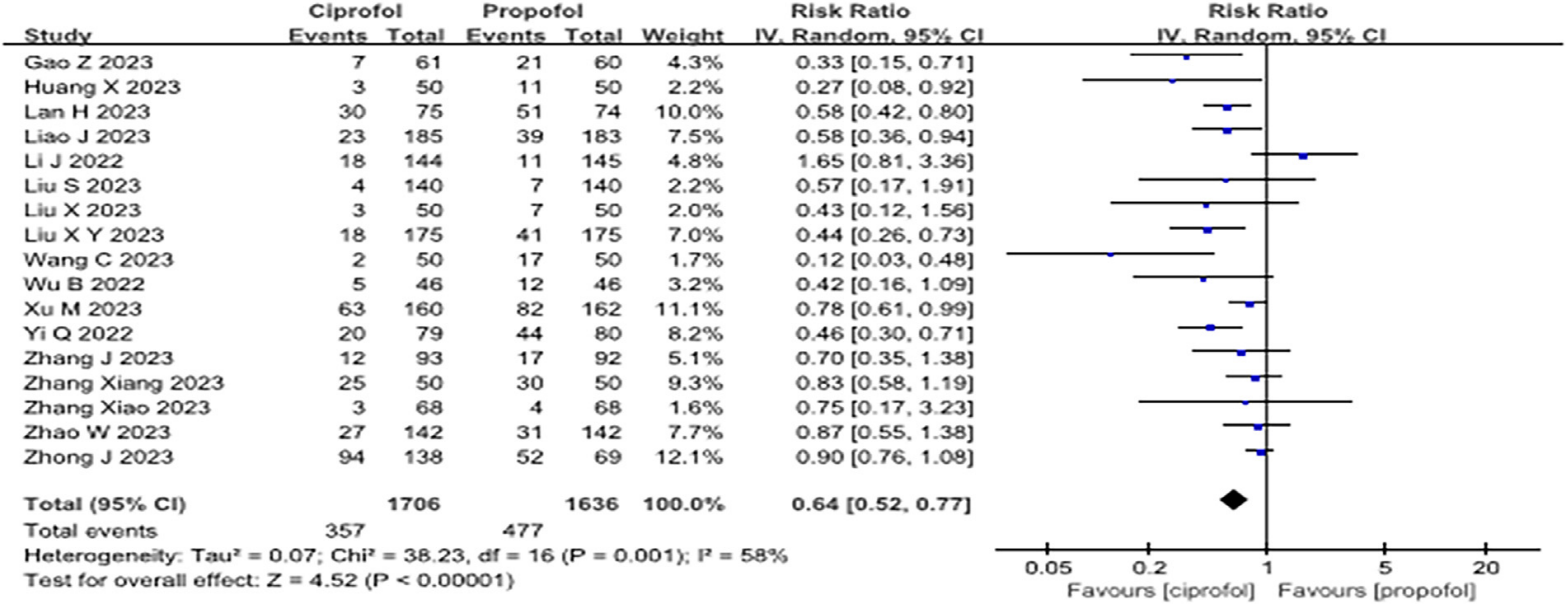
Forest plot of the incidence of hypotension during sedative sedation. "IV, Random" indicates that the inverse variance method under a random-effects model was used. When the number of included studies exceeded 4, this traditional random-effects model was applied to pool effect sizes.

Seventeen of the included RCTs^10^^,^^11^^,^^29^, ^30^, ^31^^,^^33^, ^34^, ^35^, ^36^, ^37^^,^^39^, ^40^, ^41^, ^42^, ^43^, ^44^, ^45^ reported the effect of ciprofol on the incidence of perioperative hypotension, 4 studies^11^^,^^31^^,^^39^^,^^42^ reported on the incidence of hypertension, and 14 studies^10^^,^^11^^,^^29^^,^^31^^,^^34^, ^35^, ^36^, ^37^^,^^39^, ^40^, ^41^, ^42^, ^43^, ^44^ evaluated the incidence of bradycardia with ciprofol during sedation. Compared to propofol, ciprofol significantly reduced the incidence of intraoperative hypotension (RR = 0.64; 95% CI 0.52–0.77, p < 0.001, I^2^ = 58%, low quality, Fig. 5 and Supplementary Table 1). However, the incidence of intraoperative hypertension (RR = 1.00; 95% CI 0.34–2.93, p = 1.00, I^2^ = 0, low quality, Supplementary Fig. 3 and Table 1) and bradycardia (RR = 0.84; 95% CI 0.61–1.16, p = 0.28, I^2^ = 45%, moderate quality, Supplementary Fig. 4 and Table 1) did not differ between ciprofol and propofol.

#### Nausea and vomiting

Eleven studies^27^^,^^29^, ^30^, ^31^^,^^33^^,^^35^, ^36^, ^37^^,^^40^, ^41^, ^42^ investigated the occurrence of nausea and vomiting during sedation. Patients who received ciprofol experienced a lower frequency of nausea and vomiting than those receiving propofol (RR = 0.67; 95% CI 0.49–0.92, p = 0.01, I^2^ = 0%, moderate quality; Supplementary Fig. 5 and Table 1).

#### Postoperative awakening time

Nineteen articles^10^^,^^27^, ^28^, ^29^, ^30^, ^31^, ^32^^,^^34^, ^35^, ^36^, ^37^, ^38^, ^39^, ^40^, ^41^, ^42^, ^43^, ^44^, ^45^ reported the postoperative awake times of patients with sedation induced with ciprofol. No differences in postoperative awakening time were observed between the patients receiving ciprofol or propofol (mean difference: 0.44; 95% CI -0.17 to 1.05 min, p = 0.16, I^2^ = 96%; low quality; Supplementary Fig. 6 and Table 1).

## Discussion

Our study revealed that the new sedative drug ciprofol may provide advantages over the widely used propofol in terms of reducing adverse respiratory events, perioperative hypotension, injection pain, and nausea and vomiting. Additionally, ciprofol appears to be comparable to propofol regarding postoperative awakening time. These findings highlight the potential broader application of ciprofol for sedation.

Regarding adverse respiratory events during sedation, ciprofol appears to offer improvements over propofol. It has been speculated that ciprofol may reduce respiratory depression in the central nervous system and airway collapse.^10^ Ciprofol also has a milder effect on swallowing function and the medullary center than propofol,^33^ which may contribute to its reduced effect on the respiratory system. Several studies have highlighted the advantages of ciprofol in managing respiratory outcomes.^8^^,^^33^ Furthermore, the literature we reviewed consistently agrees that the sedative effect of ciprofol is at least comparable to that of propofol, with eight studies suggesting that ciprofol may even be more effective. Existing literature suggests that ciprofol provides superior sedation than propofol in painless gastrointestinal endoscopy and intensive care unit settings, with a significantly lower incidence of adverse reactions, such as bradycardia and hypotension.^8^ Based on our analysis, we can conclude that we could not detect significantly increased cardiovascular or respiratory adverse events associated with ciprofol.

Propofol can cause hemodynamic fluctuations, mainly because it inhibits sympathetic nerve activity and decreases peripheral vascular resistance.^39^^,^^46^ A prospective cohort study of gastrointestinal endoscopy with propofol, where 98.5% of patients were administered propofol for sedation, revealed that the incidence of significant hypotensive events (systolic blood pressure < 90 mmHg or pharmacological intervention) was approximately 11%. The study also reported that the incidence of emergency events (including significant airway obstruction, hypoxia, hypotension, bradycardia, and unplanned tracheal intubation) was approximately 17%.^47^ However, ciprofol has weaker effects than propofol on myocardial contractility and peripheral vascular dilation, which may contribute to a more stable hemodynamic environment for patients during procedures.^33^^,^^48^ Notably, hypotension and respiratory events, do have significant clinical meaning. Even though these events are statistically significant (p < 0.05), they represent critical safety concerns in clinical practice, and their management can substantially impact patient outcomes.^49^^,^^50^

In terms of injection pain, the advantage of ciprofol is clear. Injection pain affects the comfort of medical procedures for patients, and rhabdomyolysis caused by infusion of a large amount of the drug is a concern for clinicians.^46^^,^^51^ One study reported that the incidence of injection pain was as high as 30%–90%,^52^ which could be because propofol is insoluble in water. Therefore, it is formulated as a 10% solution in a fat emulsion containing 1% soybean oil, which consists of long-chain triglycerides that can cause pain upon injection.^53^ However, ciprofol is water-soluble and prepared in an oil-in-water emulsion. Interestingly, its elevated hydrophobicity and reduced plasma concentration contribute to a reduction in injection pain.^27^

Our analysis suggests that ciprofol is a promising alternative in sedative procedures. Recent meta-analyses have compared the advantages of ciprofol and propofol in elective surgeries and sedation.^54^, ^55^, ^56^, ^57^ However, these studies have not specifically addressed the advantages of ciprofol concerning adverse respiratory events compared to propofol. Additionally, previous studies had more heterogeneous populations and types of surgeries than ours, which specifically included patients with ASA physical status classifications IV–V and involved both sedative and non-cardiac surgeries, providing a more targeted analysis of the benefits of ciprofol.

Our study has some limitations. First, most of the included studies had an unclear or high risk of bias, which downgraded the quality of the evidence and underscores the need for further high-quality research. Second, as most of the studies were conducted in China, the generalizability of our findings to other populations is limited. Third, since the majority of the procedures were gastrointestinal, further research is required to evaluate ciprofol's effectiveness in more invasive, high-risk surgeries. Finally, there was heterogeneity among the study designs, with variations in combined drug use, patient age, and surgery types contributing to this. Although we performed subgroup and sensitivity analyses, some unreported sources of heterogeneity could not be fully explored.

## Conclusion

Our meta-analysis suggests that ciprofol may offer advantages over propofol in reducing the frequency of perioperative adverse respiratory events and maintaining hemodynamic stability during sedation; however, caution is necessary when interpreting these results due to the low quality of the available evidence. Therefore, high-quality studies are required to make more definitive comparisons between these drugs and confirm these findings.

## Ethics approval and consent to participate

Not applicable.

## Consent for publication

Not applicable.

## Availability of data and materials

The datasets used and analyzed during the current study are available from the corresponding author upon reasonable request.

## Authors’ contributions

JZQ, LJZ, FHM, XZ and MQL contributed to the study conception and design. MQL, LQG, XZ and XYY acted as investigators or regional investigators and contributed to the acquisition of data. The analysis and interpretation of data were performed by JZQ. FHM, LJZ, XZ, BXC and MQL drafted the manuscript. MQL is responsible for the overall content as guarantor. All authors were involved in critically revising the work for important intellectual content and approval of the final manuscript. XZ and MQL attest that all listed authors meet the authorship criteria and that no others meeting the criteria have been omitted.

## Funding

This work was supported by the National Natural Science Foundation, People's Republic of China (grant numbers 82371286 and 82101350) to Mengqiang Luo.

## Conflicts of interest

The authors declare no conflicts of interest.

## Appendix Supplementary materials

Image, application 1

## Footnotes

Supplementary material associated with this article can be found in the online version at doi:10.1016/j.bjane.2024.844578.
