# Supplementary material for: Comparative effects of ciprofol and propofol on perioperative outcomes: a systematic review and meta-analysis of randomized controlled trials
Source: Braz J Anesthesiol. 2024 Nov 26;75(2):844578. doi: 10.1016/j.bjane.2024.844578 (PMC11699592; doi:10.1016/j.bjane.2024.844578)
Supplement: Supplementary file 1 [file mmc1.docx]

**BJAN-D-24-00333_Supplementary Material**

**Supplementary Table 1** Quality of evidence for primary outcome and secondary outcome.

| **Comparison** | **Number of studies, (sample size^a^)** | **Required information size met**^b^ | **Mean difference/Risk Ratio between two groups [95% CI]**^c^ | **Percentage of high or unclear ROB**^d^ | **Egger’s test (p-value)** | **I^2^**^c^ | **Quality of evidence^e^** | **Comments^e^** |
| --- | --- | --- | --- | --- | --- | --- | --- | --- |
| Respiratory adverse events | 20 (3750) | Yes | 0.44 [0.35, 0.55] | 70.0% | 0.003 | 45% | Low | Imprecision ☒ |
|  |  |  |  |  |  |  |  | Indirectness ☒ |
|  |  |  |  |  |  |  |  | Inconsistency ☒ |
|  |  |  |  |  |  |  |  | Study limitations (ROB) ☐^g^ |
|  |  |  |  |  |  |  |  | Publication bias ☐^i^ |
| Hypotension | 17 (3342) | Yes | 0.64 [0.52, 0.77] | 70.5% | 0.018 | 58% | Low | Imprecision ☒ |
|  |  |  |  |  |  |  |  | Indirectness ☒ |
|  |  |  |  |  |  |  |  | Inconsistency ☒ |
|  |  |  |  |  |  |  |  | Study limitations (ROB) ☐^g^ |
|  |  |  |  |  |  |  |  | Publication bias ☐^i^ |
| Hypertension | 4 (633) | No | 1.00 [0.34, 2.93] | 75.0% | N/A | 0% | Low | Imprecision ☐^h^ |
|  |  |  |  |  |  |  |  | Indirectness ☒ |
|  |  |  |  |  |  |  |  | Inconsistency ☒ |
|  |  |  |  |  |  |  |  | Study limitations (ROB) ☐^g^ |
|  |  |  |  |  |  |  |  | Publication bias ☐^i^ |
| Bradycardia | 14 (2590) | Yes | 0.84 [0.61, 1.16] | 71.4% | 0.688 | 45% | Moderate | Imprecision ☒ |
|  |  |  |  |  |  |  |  | Indirectness ☒ |
|  |  |  |  |  |  |  |  | Inconsistency ☒ |
|  |  |  |  |  |  |  |  | Study limitations (ROB) ☐^g^ |
|  |  |  |  |  |  |  |  | Publication bias ☒ |
| Injection pain | 20 (3746) | Yes | 0.12 [0.08, 0.17] | 70.0% | 0.038 | 36% | Low | Imprecision ☒ |
|  |  |  |  |  |  |  |  | Indirectness ☒ |
|  |  |  |  |  |  |  |  | Inconsistency☒ |
|  |  |  |  |  |  |  |  | Study limitations (ROB) ☐^g^ |
|  |  |  |  |  |  |  |  | Publication bias☐^i^ |
| Perioperative nausea and vomiting | 11 (2103) | Yes | 0.67 [0.49, 0.92] | 72.7% | 0.148 | 0% | Moderate | Imprecision ☒ |
|  |  |  |  |  |  |  |  | Indirectness ☒ |
|  |  |  |  |  |  |  |  | Inconsistency ☒ |
|  |  |  |  |  |  |  |  | Study limitations (ROB) ☐^g^ |
|  |  |  |  |  |  |  |  | Publication bias ☒ |
| Time to awakening after surgery | 19 (3271) | Yes | 0.44 [-0.17, 1.05] | 78.9% | 0.252 | 96% | Low | Imprecision ☒ |
|  |  |  |  |  |  |  |  | Indirectness ☒ |
|  |  |  |  |  |  |  |  | Inconsistency ☐^f^ |
|  |  |  |  |  |  |  |  | Study limitations (ROB) ☐^g^ |
|  |  |  |  |  |  |  |  | Publication bias ☒ |

CI, Confident Interval; ROB, Risk of Bias; N/A, Not Applicable; GRADE, Grading of Recommendations Assessment, Development, and Evaluation.

^a^ The sample size is the sum of patients included in the body of evidence.

^b^ Required information size was evaluated according to GRADE guidelines 6.[1]

^c^ The mean difference, its 95% CI, and I^2^ were calculated based on the pairwise meta-analysis.

^d^ Calculated as the number of studies with high ROB divided by the number of studies in the body of evidence. The details of the assessment of ROB are presented in figure SF1.

^e^ The evidence quality was graded according to the GRADE guidelines.[2]

^f^ Downgraded one level for inconsistency as a result of high statistical heterogeneity (I^2^ ≥ 60%) that could not be explained by subgroup analyses.[3]

^g^ Downgraded one level for study limitations (the percentage of studies with a high overall ROB ≥ 50%).[4]

^h^ Downgraded one level for imprecision because the required information size was not met.[1]

^i^ Downgraded one level for publication bias. The publication bias was determined by visual inspection of the funnel plots, with asymmetry suggesting the existence of publication bias.

**Supplementary Table 2** “Leave-one-out” sensitivity analyses of the incidence of adverse respiratory events.

| **Study** | **Risk ratio** | **95% CI lower limit** | **95% CI upper limit** | **z-value** | **I^2^** | **p value** |
| --- | --- | --- | --- | --- | --- | --- |
| Chen L 2023 | 0.44 | 0.35 | 0.55 | 7.12 | 48% | p < 0.05 |
| Gao Z 2023 | 0.44 | 0.36 | 0.56 | 7.07 | 47% | p < 0.05 |
| Huang X 2023 | 0.45 | 0.37 | 0.56 | 7.19 | 42% | p < 0.05 |
| Lan H 2023 | 0.46 | 0.38 | 0.57 | 7.23 | 38% | p < 0.05 |
| Liang W 2023 | 0.42 | 0.34 | 0.53 | 7.43 | 45% | p < 0.05 |
| Liao J 2023 | 0.42 | 0.33 | 0.52 | 7.57 | 32% | p < 0.05 |
| Li J 2022 | 0.43 | 0.34 | 0.54 | 7.14 | 48% | p < 0.05 |
| Liu S 2023 | 0.44 | 0.35 | 0.55 | 7.06 | 48% | p < 0.05 |
| Liu X 2023 | 0.44 | 0.35 | 0.55 | 7.01 | 47% | p < 0.05 |
| Liu X Y 2023 | 0.46 | 0.37 | 0.57 | 6.94 | 41% | p < 0.05 |
| Wang C 2023 | 0.45 | 0.36 | 0.56 | 7.08 | 45% | p < 0.05 |
| Wang J 2023 | 0.44 | 0.35 | 0.55 | 7.14 | 48% | p < 0.05 |
| Wu B 2022 | 0.42 | 0.33 | 0.53 | 7.36 | 46% | p < 0.05 |
| Xu M 2023 | 0.43 | 0.34 | 0.55 | 6.87 | 48% | p < 0.05 |
| Yi Q 2022 | 0.47 | 0.38 | 0.58 | 7.20 | 35% | p < 0.05 |
| Zhang J 2023 | 0.43 | 0.34 | 0.54 | 7.15 | 48% | p < 0.05 |
| Zhang Xiao 2023 | 0.42 | 0.33 | 0.54 | 6.72 | 48% | p < 0.05 |
| Zhang Xiang 2023 | 0.45 | 0.35 | 0.56 | 6.94 | 46% | p < 0.05 |
| Zhao W 2023 | 0.44 | 0.35 | 0.55 | 7.05 | 48% | p < 0.05 |
| Zhong J 2023 | 0.43 | 0.34 | 0.54 | 7.14 | 48% | p < 0.05 |

**Supplementary Figure 1** Funnel plot of the incidence of adverse respiratory events.

**
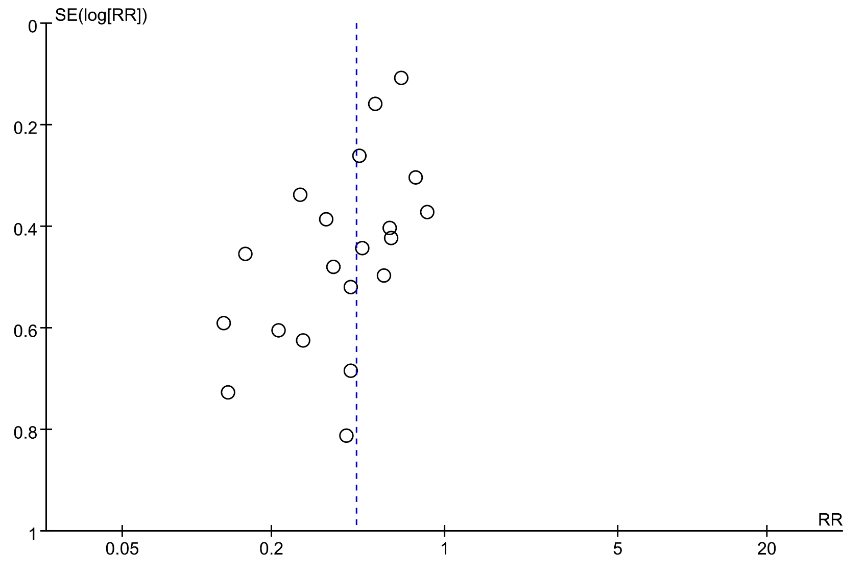
**

**Supplementary Figure 2** Forest plot of the incidence of adverse respiratory events after excluding high risk-of-bias studies. "IV, Random" indicates that the inverse variance method under a random-effects model was used. When the number of included studies exceeded 4, this traditional random-effects model was applied to pool effect sizes.


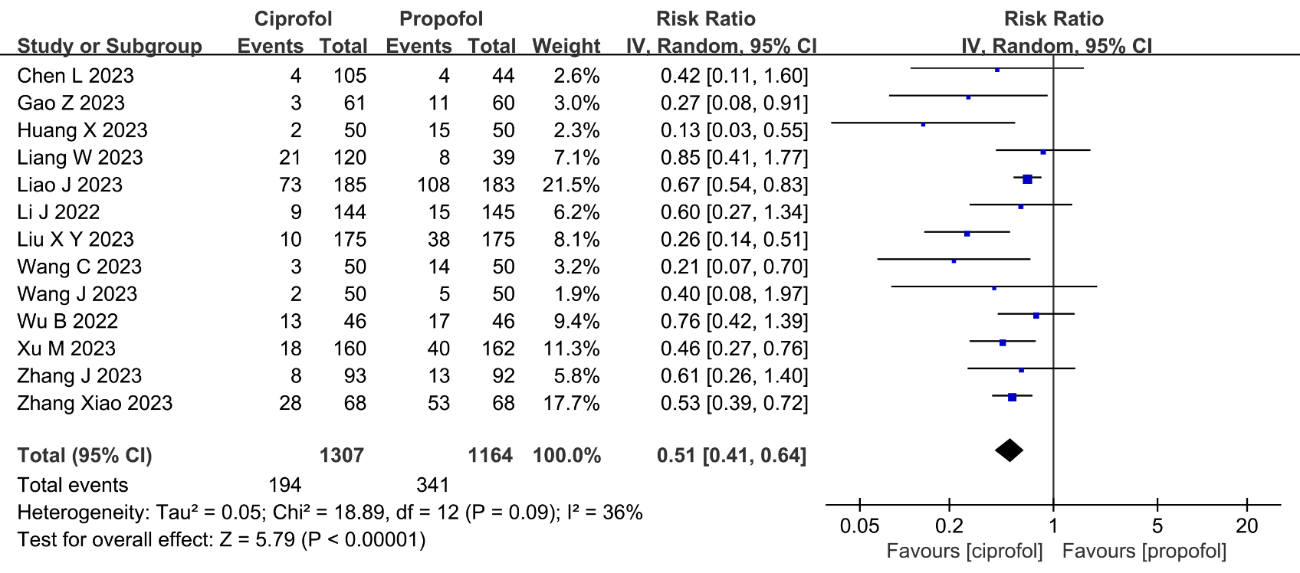


**Supplementary Figure 3** Forest plot of the incidence of hypertension. This analysis of the incidence of hypertension used a random-effects model with the Hartung-Knapp-Sidik-Jonkman (HKSJ) adjustment. When the number of included studies fewer than 5, HKSJ-adjusted random-effects model was applied to pool effect sizes.

**
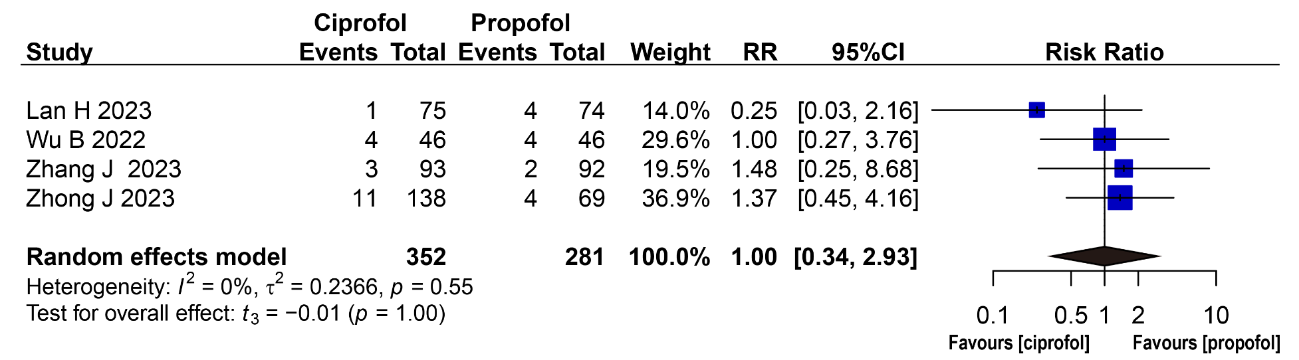
**

**Supplementary Figure 4** Forest plot of the incidence of bradycardia. "IV, Random" indicates that the inverse variance method under a random-effects model was used. When the number of included studies exceeded 4, this traditional random-effects model was applied to pool effect sizes.


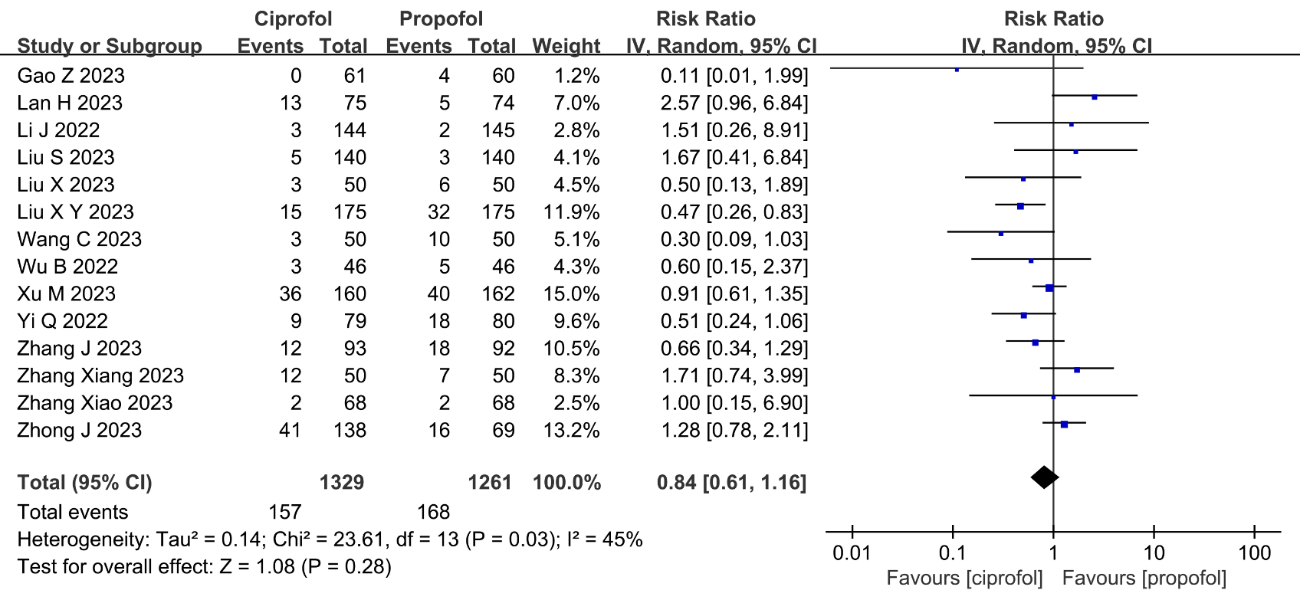


**Supplementary Figure 5** Forest plot of the perioperative nausea and vomiting. "IV, Random" indicates that the inverse variance method under a random-effects model was used. When the number of included studies exceeded 4, this traditional random-effects model was applied to pool effect sizes.


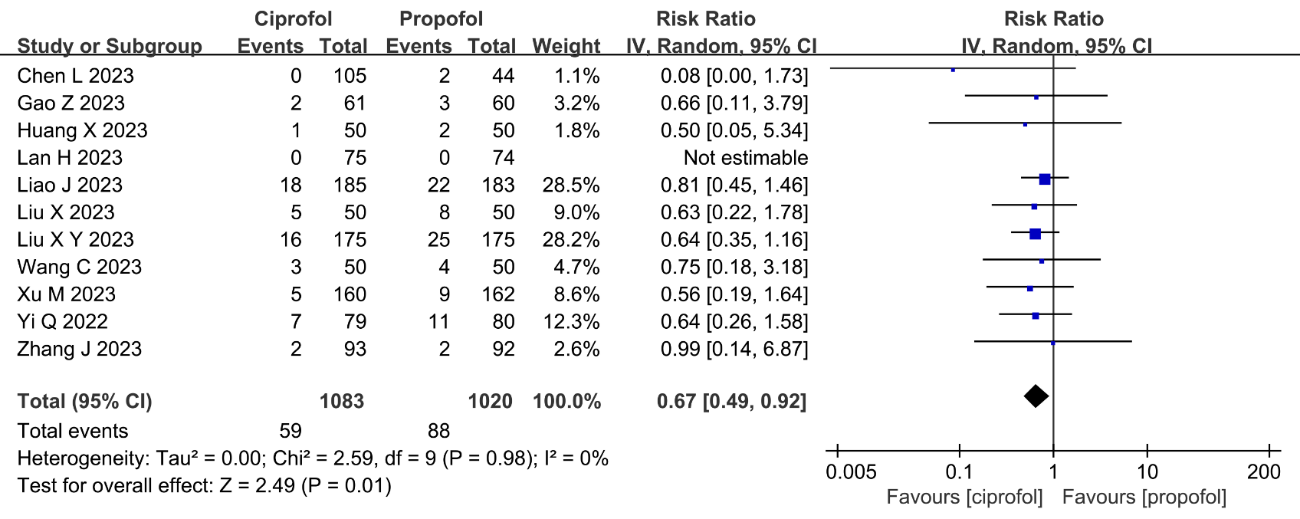


**Supplementary Figure 6** Forest plot of the time to awakening after surgery. "IV, Random" indicates that the inverse variance method under a random-effects model was used. When the number of included studies exceeded 4, this traditional random-effects model was applied to pool effect sizes.


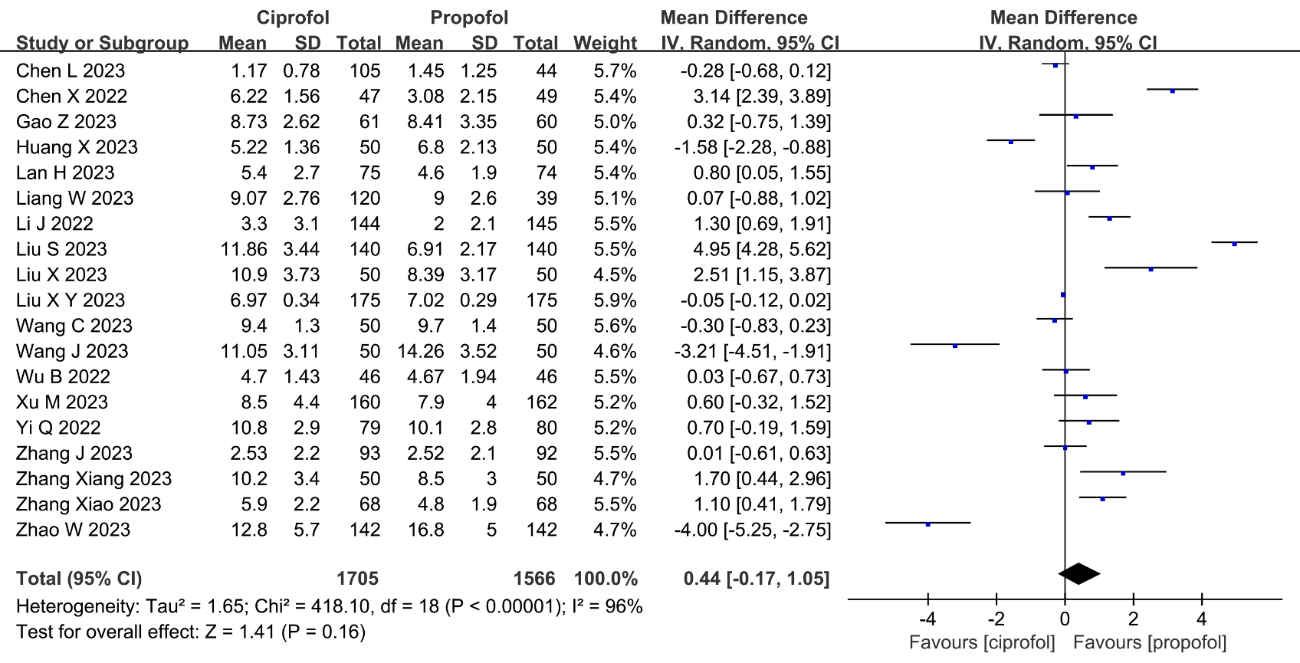


**Supplementary Figure 7** The subgroup analysis of adverse respiratory events related to short or long-acting opioids. "IV, Random" indicates that the inverse variance method under a random-effects model was used. When the number of included studies exceeded 4, this traditional random-effects model was applied to pool effect sizes.


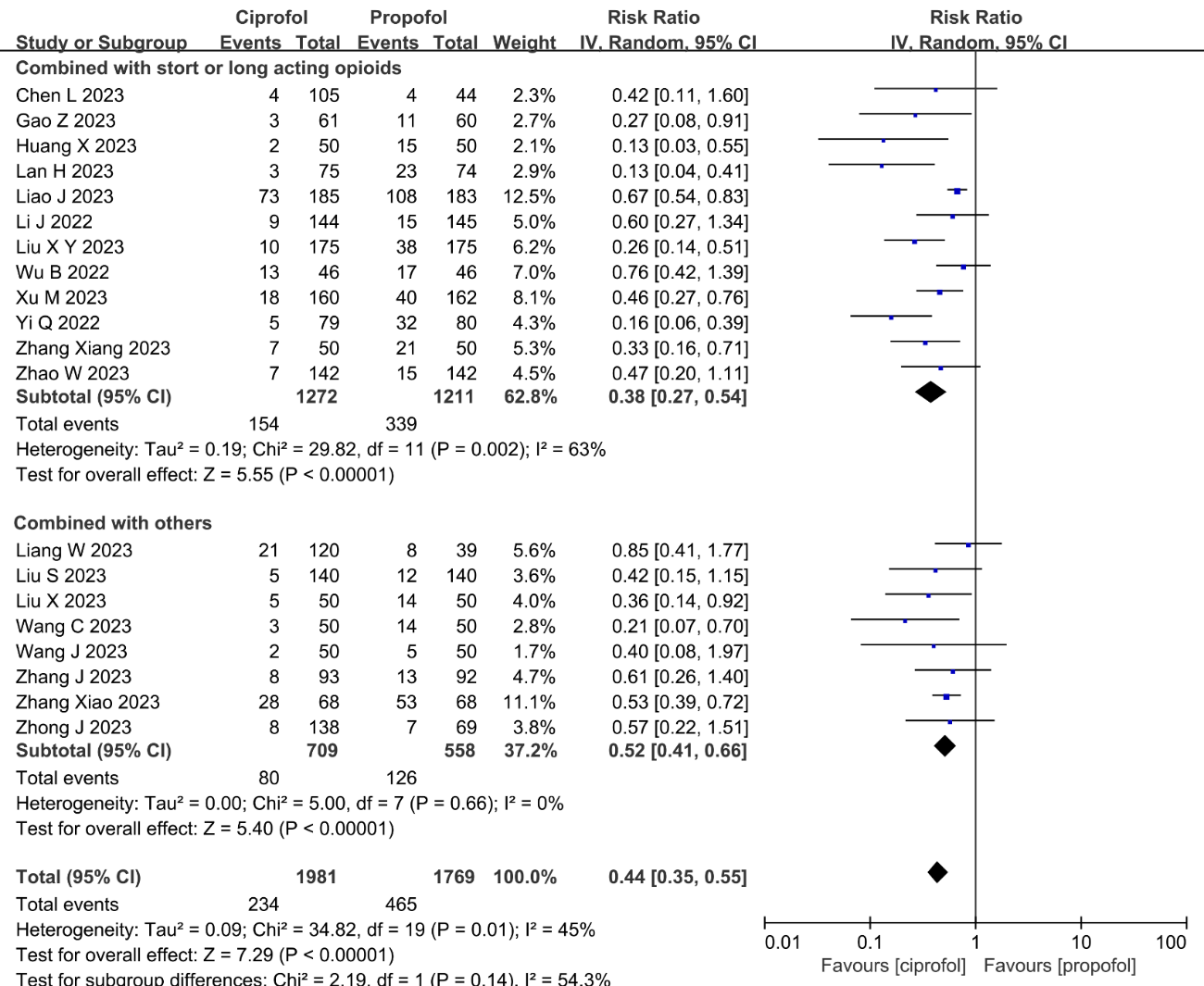


**Supplementary Figure 8** The subgroup analysis of adverse respiratory events related to surgery type. "IV, Random" indicates that the inverse variance method under a random-effects model was used. When the number of included studies exceeded 4, this traditional random-effects model was applied to pool effect sizes.


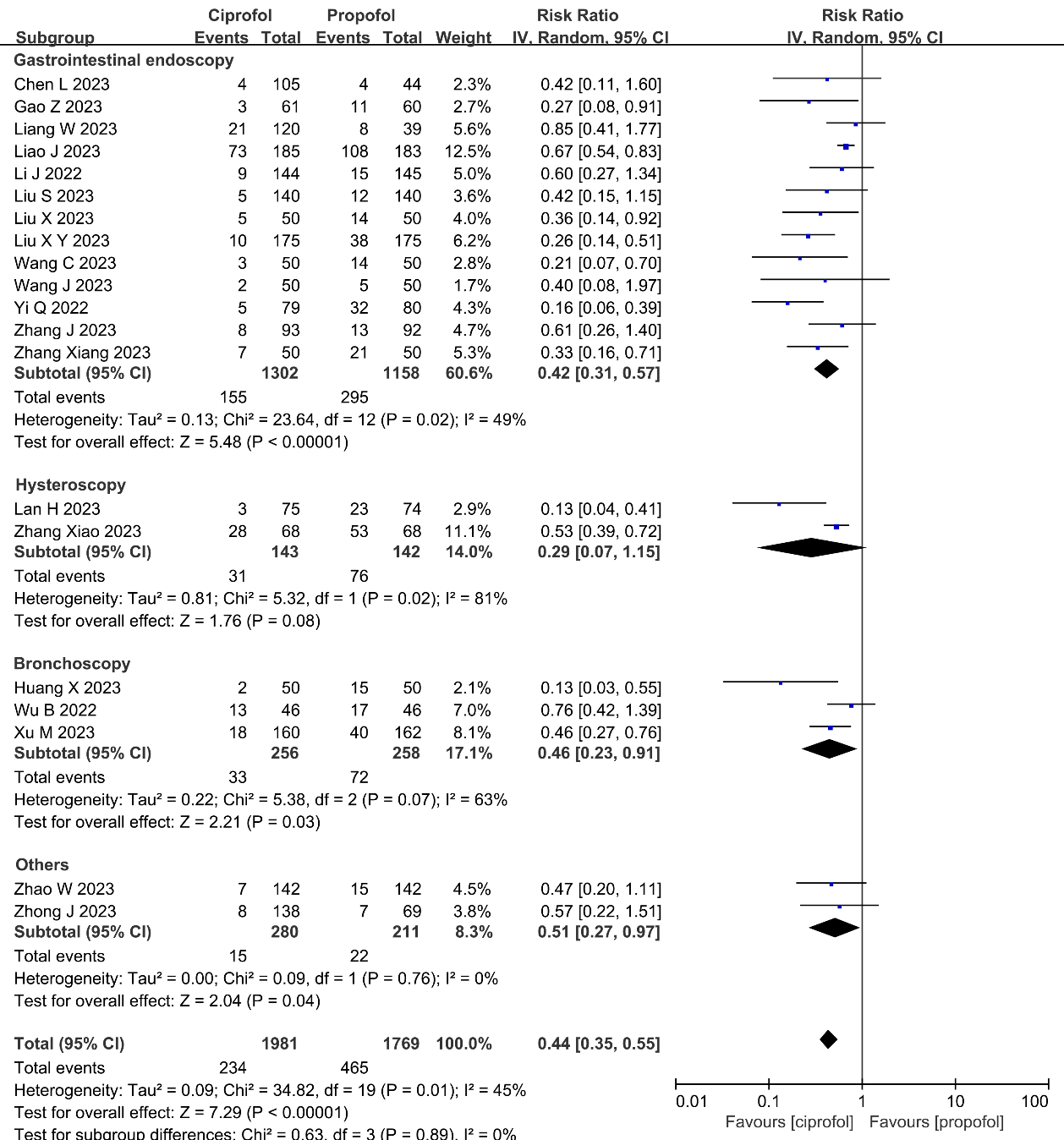


**Appendix A** Supplementary data.

**Appendix 1** Search strategies.

| **Foreign Language Database** | **Search concept** | **Results** |
| --- | --- | --- |
| Pubmed (November 27, 2023) | ciprofol" [All Fields] " | 45 |
| Web of science (November 27, 2023) | ciprofol (Topic) | 57 |
| Cochrane (November 27, 2023) | (ciprofol):ti,ab,kw | 171 |

| **Chinese Language Database** | **Search concept** | **Results** |
| --- | --- | --- |
| Wangfang (November 27, 2023) | 题名或关键词: 环泊酚 | 777 |
| Weipu (November 27, 2023) | 题名/关键词 = 环泊酚 | 136 |

**Reference**

1. Guyatt GH, Oxman AD, Kunz R, Brozek J, Alonso-Coello P, Rind D, et al. GRADE guidelines 6. Rating the quality of evidence ‒ imprecision. Journal of clinical epidemiology. 2011;64(12):1283-93.

2. Guyatt G, Oxman AD, Akl EA, Kunz R, Vist G, Brozek J, et al. GRADE guidelines: 1. Introduction-GRADE evidence profiles and summary of findings tables. J Clin Epidemiol. 2011 Apr;64(4):383-94. PubMed PMID: 21195583. Epub 2011/01/05. eng.

3. Guyatt GH, Oxman AD, Kunz R, Woodcock J, Brozek J, Helfand M, et al. GRADE guidelines: 7. Rating the quality of evidence ‒ inconsistency. Journal of clinical epidemiology. 2011;64(12):1294-302.

4. Higgins JP, Green S. Cochrane handbook for systematic reviews of interventions: John Wiley & Sons; 2011.
